# Arabic Digits-in-Noise Tests: Relations to Hearing Loss and Comparison of Diotic and Antiphasic Versions

**DOI:** 10.1177/23312165251320439

**Published:** 2025-03-21

**Authors:** Adnan M. Shehabi, Christopher J. Plack, Margaret Zuriekat, Ola Aboudi, Stephen A. Roberts, Joseph Laycock, Hannah Guest

**Affiliations:** 1 Department of Audiology and Speech Therapy, 37769 Birzeit University, Birzeit, Palestine; 2 Manchester Centre for Audiology and Deafness, 5292 The University of Manchester, Manchester, UK; 3 Psychology Department, 4396 Lancaster University, Lancaster, UK; 4 Special Surgery Department, School of Medicine, 54658 The University of Jordan and Jordan University Hospital, Amman, Jordan; 5 Centre for Biostatistics, 5292 The University of Manchester, Manchester, UK; 6 272245 Ansys Inc., Canonsburg, Pennsylvania, USA

**Keywords:** Digits-In-Noise, Digit Triplet Test, Arabic, hearing screening, speech-in-noise

## Abstract

The study set out to acquire validation data for Arabic versions of the Digits-in-Noise (DIN) test, measured using browser-based software suitable for home hearing screening. DIN and pure-tone audiometric (PTA) thresholds were obtained from a sample of 155 Arabic-speaking participants, varying widely in age and in degree and type of hearing loss. DIN thresholds were measured using both diotic and antiphasic stimuli, with the goal of determining whether antiphasic testing provides superior prediction of poorer-ear hearing loss. A comprehensive study protocol was publicly pre-registered via the Open Science Framework. Both types of DIN threshold correlate with poorer-ear PTA thresholds after controlling for age, but the correlation is significantly stronger for antiphasic than diotic stimuli. Antiphasic DIN thresholds increase more steeply than diotic DIN thresholds as poorer-ear PTA thresholds increase, and are superior binary classifiers of hearing loss. Combined with previous results based on DIN data measured in participants’ homes, the present findings suggest that the browser-based Arabic DIN test may be effective in remote hearing screening, when combined with antiphasic digit presentation.

## Introduction

The World Health Organization estimates that half a billion people suffer from disabling hearing impairment, and that the burden of unaddressed hearing loss is greatest in low- and middle-income countries, including many Arabic-speaking Middle Eastern and North African nations (World Health Organization, 2018; 2023). Limited access to hearing healthcare in these regions is a crucial contributor (World Health Organization, 2013), suggesting an important role for remote hearing screening. Hindering remote screening efforts is the scarcity of Arabic-language test materials, whose development lags far behind those in European languages (Alsabbagh et al., 2020; Kishon-Rabin & Rosenhouse, 2000), despite Arabic's 400 million native speakers worldwide (Central Intelligence Agency, 2018).

The Digits-In-Noise (DIN) test (Smits et al., 2004) is used for hearing screening in a variety of world languages (Van den Borre et al., 2021). The test assesses listeners’ ability to recognise simple words (digits in the range 0 to 9) in the presence of a masker - often speech-shaped noise - and typically yields steep psychometric functions and precise threshold measurements (Van den Borre et al., 2021). Originally developed in Dutch for hearing screening by landline telephone (Smits et al., 2004), the DIN test has since been delivered via mobile communication networks, the internet, tablets, computers, and smartphones. It has been translated into at least 15 languages (Van den Borre et al., 2021) and the World Health Organization's hearWHO app has been used over a quarter of a million times (De Sousa et al., 2022a).

Recently developed online DIN software (Shehabi et al., 2024) allows remote testing in Arabic via the listener's web browser and headphone/earphones, without the need to install an app. Arabic DIN thresholds measured using this software have been shown to relate to ageing and noise exposure (Shehabi et al., 2023) but thorough validation is required before the software can be used to screen for hearing loss. Such validation data may also be valuable to researchers and clinicians concerned with DIN methods, especially in the Arabic-speaking world.

Preliminary validation of the browser-based Arabic DIN test (Shehabi et al., 2024) investigated the effects of test language (Arabic versus English) and test environment (lab versus home). In a sample of 52 bilingual listeners with normal hearing, DIN thresholds were slightly but significantly higher for Arabic stimuli (mean = −10.75 dB, SD = 0.92 dB) than English stimuli (mean = −11.49 dB, *SD* = 1.10 dB). Test environment had a more marginal effect than test language: DIN thresholds obtained in the lab were ∼0.5 dB lower than those obtained at home using the listeners’ own equipment, though the effect did not survive correction for multiple comparisons. Absolute test-retest differences were low for both languages and both test environments. Results suggest that browser-based self-testing at home could yield valid and reliable Arabic DIN data. However, the authors concluded that data collection in a sample of Arabic speakers with hearing loss was necessary, in order to confirm validity and reliability, and to generate reference data for use in hearing screening.

An additional recent advance in DIN testing is the development and validation of the antiphasic DIN test, originally in South African English (De Sousa et al., 2020; De Sousa et al., 2022b) and later in French (Ceccato et al., 2021). In antiphasic presentation, the phase of the digits is inverted between the ears, while the masker remains diotic, creating a binaural advantage for normally hearing listeners. De Sousa and colleagues showed that correlations between DIN and poorer-ear pure-tone audiometric (PTA) thresholds are (after controlling for age) stronger for antiphasic than diotic stimuli. Receiver-operating-characteristic (ROC) curve analysis also revealed the superiority of the antiphasic test in detecting poorer-ear hearing loss >25 dB HL or >40 dB HL. Antiphasic DIN testing may therefore be preferable to diotic or monaural where the goal of testing is to detect poorer-ear hearing loss using a single test block. Hence, if a remote hearing screening programme aims to rapidly identify individuals with at least one ear that might benefit from audiological intervention, then the antiphasic DIN test appears valuable.

However, the question of whether these results extend to Arabic test material is unanswered. This is true not only of the antiphasic DIN test but also diotic and monaural versions, none of which have yielded published validation data relating Arabic DIN to PTA thresholds. This gap in the literature represents a crucial impediment to use of DIN-based hearing screening in the Arabic-speaking world. Arabic is fundamentally distinct from Dutch, English, and the other European languages used to establish the validity of the DIN test, due to its unique phonetic inventory, multisyllabic numeral structure, and language-specific cognitive processing demands. Digits in European languages like English, French, or German are typically monosyllabic, whereas Arabic digits are consistently multisyllabic. A number of Arabic digits share phonemic content (e.g., “thalaatha” [3] and “thamaniya” [8]). Both features potentially increase the cognitive and perceptual load in background noise. Arabic also has a unique phonetic inventory, including emphatic consonants, uvular sounds, and a distinct vowel system, which may interact differently with background noise compared to the phonetic inventories of European languages. Existing validation of the DIN in other languages cannot therefore be assumed to extend to Arabic. Koifman et al. (2016) reported preliminary Arabic DIN evaluation data in 15 listeners, but all had normal hearing and validation against PTA thresholds was not attempted. It remains essential to validate the DIN test within the unique linguistic framework of Arabic.

It is also important to note that DIN test interfaces used by speakers of European languages cannot be straightforwardly repurposed for Arabic speakers, and this may partly explain the absence of publicly available Arabic DIN materials. Arabic's right-to-left writing system necessitates extensive modifications to the user interface. These modifications go beyond merely changing text direction; they include realigning interface elements, adjusting navigation flows, redesigning interactive components, and ensuring overall usability for Arabic-speaking users. Our team has found that this process requires skilled programmers and designers who are not only familiar with psychophysical methods and fluent in Arabic but also proficient in web design and capable of addressing the complexities of right-to-left interfaces. This specialized expertise increases the resource demands of the adaptation process. Reflecting these challenges, recent reviews have noted the slower progression of telemedicine (Waqas et al., 2021) and health informatics (El Jabari & Adwan, 2019) in the Arabic-speaking world. These complexities underscore the necessity of the present validation study, as neither the auditory nor the visual components of the DIN test can be assumed to function in Arabic as they do in European languages.

The present study was therefore undertaken to determine the validity of the browser-based Arabic DIN test, by testing its capacity to predict hearing loss in Arabic speakers with a wide range of audiometric profiles. It also aimed to perform a conceptual replication of the work of De Sousa and colleagues, comparing diotic and antiphasic versions (De Sousa et al., 2020; De Sousa et al., 2022b).

## Material and Methods

### Study Design

Native Arabic-speaking adults with a broad array of audiometric profiles provided simple self-report data and underwent tympanometry, PTA, and DIN testing. Data collection took place in the audiology clinic of Jordan University Hospital, a tertiary referral centre and teaching hospital. Data were collected by two personnel: a senior clinical audiologist and an otolaryngologist also qualified in audiology.

### Participants

**Figure 1.**
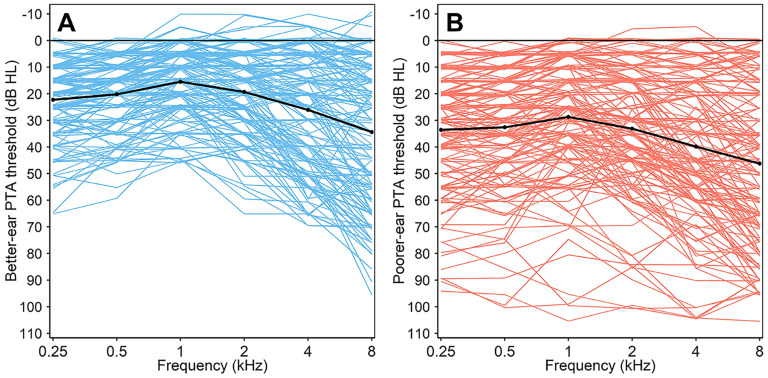
Audiometric thresholds in the better-hearing ear (A) and poorer-hearing ear (B).

A cohort of 155 eligible participants (85 female) was recruited at the University of Jordan and Jordan University Hospital, via direct approach and snowball sampling. Sources included ENT and audiology patient populations, individuals accompanying patients, hospital and university staff, and healthcare students/trainees. Eligibility criteria were deliberately broad, placing almost no restriction on type or aetiology of hearing loss. Participants were required to have at least one ear with a four-frequency pure-tone audiometric average (PTA_4FA_) < 70 dB HL, with PTA_4FA_ being defined as the arithmetic mean of the thresholds at 0.5, 1, 2, and 4 kHz. They were also required to be native speakers of Arabic, and to complete all measures required for analysis (i.e., questionnaire, PTA, and DIN testing). This permissive approach led to a sample with a wide array of audiometric profiles (Figure 1). Table 1 summarises participant characteristics.

**Table 1.** Participant characteristics. Tympanogram classifications are defined by Jerger (1970). Hearing-loss types are defined by De Sousa et al. (2022b)

Sex	Male			Female	
	n = 70			n = 85	
Age	Mean		Median		Range
	46.1 (SD = 18.8) years		46.0 years		18–83 years
Tympanogram classification	Type A	Type A-S	Type A-D	Type B	Type C
	n = 74	n = 9	n = 42	n = 27	n = 3
Type of hearing loss	Conductive		Unilateral/asymmetric	Sensorineural	Normal hearing
	n = 28		n = 15	n = 56	n = 56

One participant's data was identified as an outlier and excluded from all analyses. This listener's binaural intelligibility level difference (BILD; Licklider, 1948) was −6.5 dB, i.e., their antiphasic DIN threshold was 6.5 dB poorer than their diotic DIN threshold. This result suggests data-collection failure. No other participant had a BILD below −4.3 dB (i.e., more than three standard deviations below the mean for the sample), so this participant alone was excluded as an outlier. For the sake of transparency, we report in *Supplementary Material* the results of our analyses with this participant's data included (the pattern of results is unaltered.)

### Self-Report

Self-report data were obtained by interview and recorded on paper by the researcher (see “Clinical and Demographic Questionnaire” in *Supplementary Material*). The questionnaire included demographic data (age, sex, and highest level of educational attainment) and whether or not the participant experienced fluctuating hearing loss, vertigo, diagnosed memory problems, and current or recent ear infections.

### Pure-Tone Audiometry

PTA thresholds were measured bilaterally, in a double-walled sound-attenuating booth. Air-conduction (AC) thresholds at 0.25, 0.5, 1, 2, 4, and 8 kHz were obtained using an Interacoustics AC40 clinical audiometer and DD45 supra-aural headphones. Bone-conduction (BC) thresholds were obtained at 0.5, 1, and 2 kHz using a B81 bone conduction vibrator. All testing was conducted in accordance with British Society of Audiology recommended procedures, with masking where necessary. Any threshold that exceeded the maximum output of the audiometer was replaced with the output limit + 5 dB; in practice, this was the case for just 0.4% of thresholds.

For each ear, the arithmetic mean of the AC thresholds at 0.5, 1, 2, and 4 kHz was calculated, yielding the PTA_4FA_. Based on these values, the participant's “better-hearing ear” and “poorer–hearing ear” were identified, for use in statistical analysis.

### Digits-in-Noise Testing

#### Software and Equipment

DIN testing was carried out via browser-based software for delivery of online listening tasks (Shehabi et al., 2024). Participants listened to digit-triplet-in-noise stimuli (e.g., “The digits 4, 9, 2”) via DD45 supra-aural headphones connected to a Sony SVS131A12 W laptop, and entered them via mouse and on-screen number pad. Prior to testing, the DIN software provided participants with plain-language instructions, a demonstration, and subjective calibration of stimulus level (see *Stimuli*).

#### Stimuli

Digits in each triplet were sampled without replacement from the range 1 to 9 and were spoken by a female standard-Arabic talker. Each digit sound file was selected randomly from six exemplars, spoken by the same talker but with some natural variation in enunciation. The masker was a randomly selected segment of speech-spectrum-shaped Gaussian noise. Both target and masker were band-pass filtered between 0.12 and 8 kHz, since online hearing screening using the DIN test is ultimately expected to employ this passband. The removal of sound energy outside this band is intended to prevent results from being influenced by variations in the performance of consumer headphones and earphones at very low and high frequencies. However, it is worth noting that Culling et al. (2005) found little effect of varying consumer audio equipment on speech-in-noise performance, so this approach may be excessively cautious.

Stimuli were not optimised to adjust for discrepancies in digit intelligibility. Optimisation of stimuli is necessary to enhance the efficiency and precision of DIN threshold measurement (Zokoll et al., 2012). In the course of this process, psychometric functions are measured for individual digit tokens, in order to determine the SNRs required to achieve a target percentage correct. The relative levels of the digits are then adjusted to equalise their intelligibility. This process had not yet been conducted at the time of data collection for the present study, likely adding noise to the adaptive tracks. Precision of DIN threshold measurements was nonetheless expected to be sufficient to show clear relations between DIN and PTA thresholds and differentiate the performance of the diotic and antiphasic tests. However, optimisation will certainly be required prior to use of the digits for hearing screening.

To guard against loudness discomfort at very high or very low SNRs, the signal-to-noise ratio (SNR) was varied by adjusting the levels of both target and masker, while holding constant the overall root-mean-square (RMS) level of the stimuli. An appropriate stimulus level for the participant was determined via an initial subjective calibration stage. In this stage, the participant listened to a “loud” phrase and a “quiet” phrase (spoken by a female standard-Arabic talker, separated in RMS level by a fixed 25 dB) and adjusted the volume control on the computer until the “quiet” phrase was clear and the “loud” phrase was not uncomfortably loud. All subsequent stimuli were delivered at an RMS level 3 dB below that of the “loud” phrase.

Testing consisted of two blocks. No preceding practice block was provided, since test materials are ultimately intended for use in rapid hearing screening. In the diotic condition (Block 1), both digits and masker were interaurally in-phase. In the antiphasic condition (Block 2), the phase of the digits only was inverted between the ears. The latter configuration creates a binaural listening advantage, leading to reduced DIN thresholds in listeners with normal hearing (Smits et al., 2016).

#### Adaptive Procedure

Stimulus SNR was varied according to a two-down one-up stepping rule. A trial was considered correct if 2/3 or 3/3 digits were entered correctly. The starting SNR was 2 dB and the adaptive track consisted of four initial reversals (6 dB steps) and four threshold reversals (2 dB steps). Mean track length was 26.9 trials. Threshold was taken as the mean of the SNRs at the final four reversals. Visual feedback on trial correctness and ongoing performance was displayed throughout.

### Statistical Analysis

Statistical analysis was conducted using R (R Core Team, 2021). The statistical tests for RQ3 and RQ4 were conducted one-tailed, the remainder two-tailed. Results were corrected for four comparisons using the Bonferroni-Holm method, with a studywise error rate <0.05.

#### Relations Between Arabic DIN and PTA Thresholds

Potential relations between DIN and PTA thresholds were investigated via four inferential research questions (RQs). The questions and corresponding analysis models are as follows.

**RQ1 (inferential): Are Arabic diotic DIN thresholds related to poorer-ear PTA, controlling for age?** Analysed via partial correlation between diotic DIN thresholds and poorer-ear PTA_4FA_, controlling for age.

**RQ2 (inferential): Are Arabic antiphasic DIN thresholds related to poorer-ear PTA, controlling for age?** Analysed via partial correlation between antiphasic DIN thresholds and poorer-ear PTA_4FA_, controlling for age.

**RQ3 (inferential): Are Arabic antiphasic DIN thresholds more strongly related than Arabic diotic DIN thresholds to poorer-ear PTA?** Analysed via Williams’ t-test (Williams, 1959) comparing the two Fisher-transformed correlation coefficients (between diotic DIN thresholds and PTA_4FA_ and between antiphasic DIN thresholds and PTA_4FA_).

**RQ4 (inferential): Is the increase in Arabic DIN thresholds with increasing poorer-ear PTA steeper for antiphasic than diotic stimuli?** Analysed via multiple linear regression, with DIN thresholds as the outcome variable, poorer-ear PTA_4FA_ as a continuous predictor variable, test type (diotic or antiphasic) as a binary predictor variable, and an interaction term between the two predictors. The predictor of interest is the interaction term, which allows comparison of the steepness of the two slopes.

#### Arabic DIN Thresholds as Predictors of Hearing Loss

The predictive abilities of DIN thresholds were investigated via two descriptive research questions. The questions and corresponding analysis models are as follows.

**RQ5 (descriptive): How well do Arabic diotic DIN thresholds predict hearing loss?** Area under the ROC curve (AUC) quantifies the ability of diotic DIN thresholds to predict the presence of PTA_4FA_ > 15, 20, 25, and 40 dB HL.

**RQ6 (descriptive): How well do Arabic antiphasic DIN thresholds predict hearing loss?** AUC quantifies the ability of antiphasic DIN thresholds to predict the presence of PTA_4FA_ exceeding > 15, 20, 25, and 40 dB HL.

#### Exploratory Analyses

In addition to generating ROC curves as planned, we added DeLong's tests to formally compare the AUC for the diotic and antiphasic DIN. The results of this exploratory analysis are reported in the main paper.

One additional set of exploratory analyses examined relations between DIN thresholds and *better-ear* PTA. Another substituted the DIN BILD for DIN thresholds. Multiple linear regression models were constructed to explore the effects of age, sex, and educational attainment on DIN thresholds. The results of these exploratory analyses are reported in the *Supplementary Material*.

### Pre-Registration

To guard against undisclosed post-hoc data exploration (e.g., p-hacking and HARKing), a comprehensive study protocol was registered via the Open Science Framework (https://osf.io/86ge5).

All aspects of data collection and analysis were conducted in accordance with the registered protocol, with the exception of sample size. Sample size was intended to be 130, but, in practice, 155 participants were recruited. This overshoot was due to the unexpectedly enhanced recruitment needs of a related study on DIN threshold reliability (a lower proportion of participants agreed to return for a second session than was anticipated). Since DIN data were obtained from 155 participants, we have reported results from all 155. However, for the sake of transparency, we report in *Supplementary Material* the results of our analyses with the final 25 recruits to the study omitted (the pattern of findings is unaltered.)

## Results

### Relations Between Arabic DIN and PTA Thresholds

**Figure 2.**
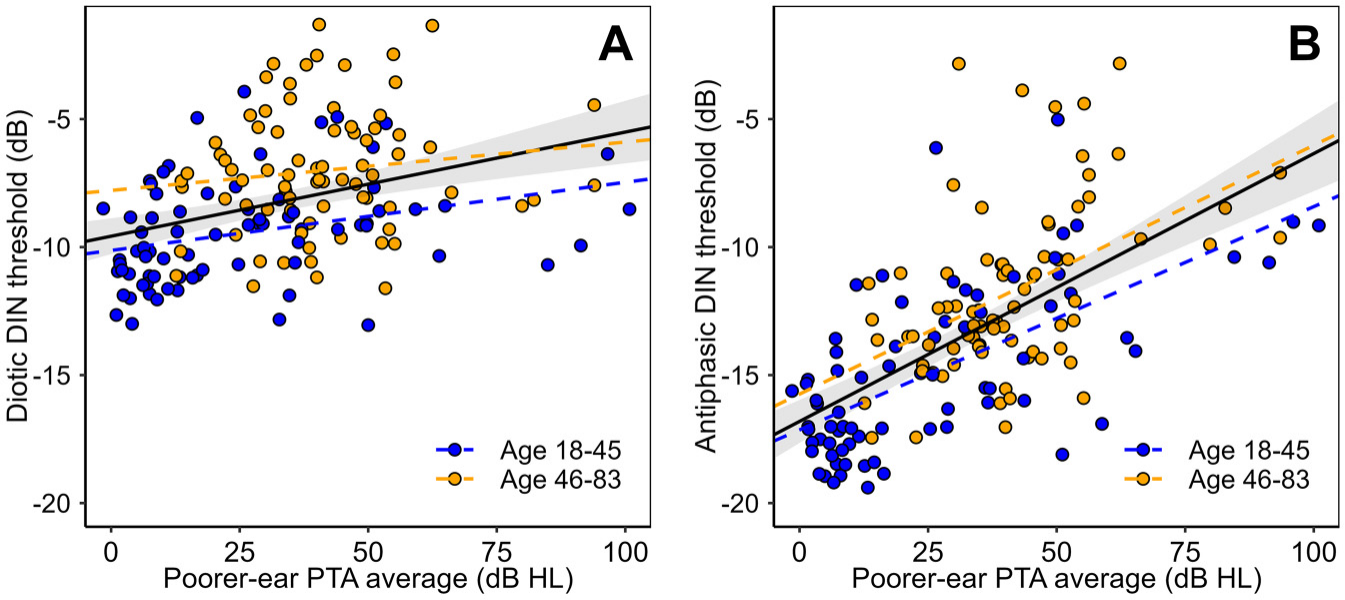
Relations between DIN thresholds and PTA_4FA_, for both diotic (A) and antiphasic (B) versions of the DIN test. The solid black lines and shaded areas represent the regression functions and 95% CIs for the full sample; the coloured lines represent the regression functions for older and younger participants.

Figure 2 illustrates relations between DIN thresholds and poorer-ear PTA_4FA_, stratified by age. Partial correlation reveals that, after controlling for age, poorer-ear PTA_4FA_ is correlated with antiphasic DIN thresholds (*r* = 0.54, *p* < 0.0001). The corresponding correlation with diotic DIN thresholds (*r* = 0.17, *p* = 0.04) does not survive correction for multiple comparisons. The correlation coefficients were compared formally using the R “cocor” package (Diedenhofen & Musch, 2015). A William's t-test confirmed greater correlation strength for antiphasic than diotic stimuli (*p* < 0.0001).

Following De Sousa et al. (2020), multiple linear regression allowed the steepness of the slopes between DIN thresholds and poorer-ear PTA_4FA_ to be compared for the two test types, diotic and antiphasic. The interaction term (test type:PTA_4FA_) is significant (*p* < 0.0001), confirming that increases in PTA_4FA_ lead to steeper increases in antiphasic DIN thresholds than in diotic DIN thresholds.

### Arabic DIN Thresholds as Predictors of Hearing Loss

**Figure 3.**
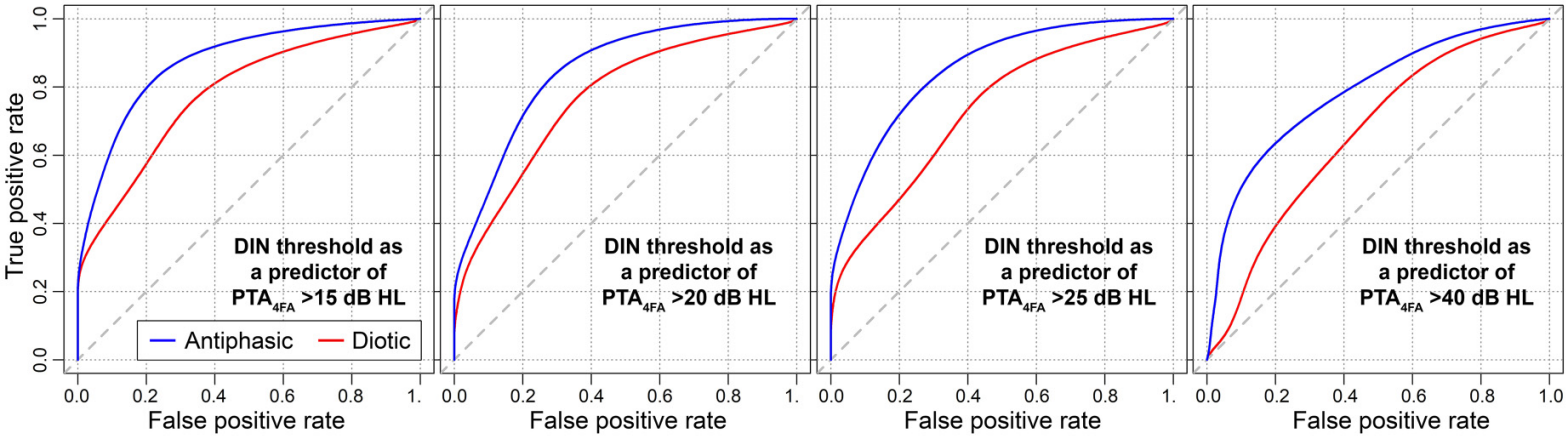
Non-parametric ROC curves illustrating the diagnostic performance of DIN thresholds in classifying various degrees of poorer-ear hearing loss.

Figure 3 shows ROC curves for the diotic and antiphasic DIN, as binary classifiers of various degrees of poorer-ear hearing loss. Table 2 contains the AUC values, along with a range of potential DIN threshold cutoffs and their corresponding sensitivities and specificities.

**Table 2.** Performance metrics of DIN thresholds for classification of poorer-ear hearing loss.

	Diotic DIN					Antiphasic DIN				
	*AUC*	*SRT cutoff (dB)*	*Sensitivity*	*Specificity*	*Youden index*	*AUC*	*SRT cutoff (dB)*	*Sensitivity*	*Specificity*	*Youden index*
Detection of poorer-ear PTA_4FA_ >15 dB HL	0.79	−8.5	0.68	0.75	0.43	0.89	−14.0	0.75	0.85	0.60
		−9.0	0.75	0.70	0.45		−14.5	0.81	0.85	0.66
		−9.5	0.82	0.65	0.47		−15.0	0.85	0.78	0.63
Detection of poorer-ear PTA_4FA_ >20 dB HL	0.78	−8.5	0.69	0.72	0.41	0.86	−14.0	0.76	0.79	0.55
		−9.0	0.78	0.68	0.46		−14.5	0.81	0.77	0.58
		−9.5	0.83	0.62	0.45		−15.0	0.86	0.70	0.56
Detection of poorer-ear PTA_4FA_ >25 dB HL	0.74	−8.5	0.69	0.65	0.34	0.87	−13.5	0.72	0.82	0.54
		−9.0	0.78	0.62	0.40		−14.0	0.79	0.76	0.55
		−9.5	0.83	0.65	0.48		−14.5	0.84	0.73	0.57
Detection of poorer-ear PTA_4FA_ >40 dB HL	0.67	−7.5	0.54	0.67	0.21	0.80	−12.0	0.65	0.82	0.47
		−8.0	0.62	0.60	0.22		−12.5	0.69	0.76	0.45
		−8.5	0.76	0.53	0.29		−13.0	0.73	0.67	0.40

As can be seen from the figure and table, AUC estimates are consistently higher for the antiphasic than the diotic DIN. As an exploratory analysis, one-tailed DeLong's tests (DeLong et al., 1988) were used to compare the AUCs formally. For all four degrees of hearing loss (PTA_4FA_ > 15, 20, 25, and 40 dB HL), the AUC is significantly higher for the antiphasic than the diotic DIN (*p* < 0.01 in all cases). These results indicate that the antiphasic DIN test is a better predictor of poorer-ear audiometric hearing loss than the diotic, performing especially well in the detection of relatively mild hearing loss.

## Discussion

### Poorer-Ear Hearing Loss is Better Predicted by Antiphasic Than Diotic DIN Thresholds

In each of the analyses involving poorer-ear audiometric thresholds, antiphasic Arabic DIN thresholds outperform diotic Arabic DIN thresholds. Their correlations with poorer-ear audiometric thresholds are stronger, they exhibit steeper regression lines with poorer-ear audiometric thresholds, and they are superior binary classifiers of poorer-ear hearing loss. These findings confirm (and extend to the Arabic language) those of De Sousa et al. (2020; 2022b), who obtained the same pattern of results using South-African-English DIN tests.

It is worth noting that corresponding results for better-ear audiometric thresholds are quite different, with the diotic DIN surpassing the antiphasic (see *Supplementary Material*). This finding echoes that of De Sousa et al. (2020) and is unsurprising, since diotic DIN performance is determined largely by the function of the better ear (Potgieter et al., 2018). Where researchers or other professionals require a DIN test reflecting better-ear hearing, the diotic DIN may be appropriate. By contrast, antiphasic stimuli appear preferable where the goal of DIN testing is to rapidly identify individuals with at least one hearing-impaired ear, as may be the case in remote hearing screening.

### Browser-Based Arabic DIN Tests Appear Suitable for Remote Hearing Screening

Despite our recruitment of an extremely diverse sample, antiphasic DIN thresholds performed well in identifying listeners with at least one ear with mild hearing loss. The software used to deliver the tests requires only a browser and headphones/earphones, so could be employed for self-testing by listeners at home. However, in the present study, testing was conducted in a sound-attenuating booth, using a single type of headphone. It is important to know whether comparable DIN thresholds are obtained in listeners’ home environments, which may differ widely in the equipment used, the levels of background noise, the presence of distractions, and listeners’ motivation in the absence of a researcher or clinician.

Shehabi et al. (2024) employed the same DIN testing software as in the present study and compared lab-based DIN thresholds with those obtained in listeners’ homes using their own equipment. Test environment had no statistically significant effect on DIN thresholds after correction for multiple comparisons, but the at-home mean was ∼0.5 dB higher than the lab-based mean, suggesting that it might be appropriate to apply a small correction to normative DIN data to account for test environment. Absolute test-retest differences were low for both environments. When combined with the present data, these results suggest that the browser-based antiphasic Arabic DIN may be a cost-effective means of screening for hearing loss, especially in regions of the Arabic-speaking world with limited access to hearing-healthcare resources.

However, an important additional step may be the adjustment of DIN threshold cutoffs for age. Of course, age is associated with increased audiometric thresholds, which in turn are associated with DIN deficits, but our data confirm an additional independent effect of age on DIN thresholds. Exploratory multiple-linear-regression analysis suggests that, after controlling for hearing loss, antiphasic DIN thresholds may increase by ∼0.5 dB per decade of life (see *Supplementary Material*). Failure to correct for this direct effect of age on DIN data might lead to false alarms in older adults and/or false misses in the young. However, it is important to acknowledge that the effects of age on DIN thresholds are likely complex, depending not only on intrinsic age effects but also age-related differences in patterns, symmetries, and aetiologies of hearing loss. In the present study, the proportion of hearing-loss cases that are bilaterally sensorineural (rather than conductive or asymmetric) increases with age, due to increasing prevalence of presbyacusis. Our sample size is not sufficient to meaningfully adjust our ROC curve analyses for age, but De Sousa et al. (2022b) were able to do so in their sample of 489. Their results did not support a requirement for age-corrected antiphasic DIN cutoff values, but the authors noted that the contribution of age to the analyses was complex, due its interplay with different types and symmetries of hearing loss. It may be worthwhile to explore the contributions of age and hearing-loss characteristics to Arabic antiphasic DIN thresholds in a larger sample.

### Limitations

Digit sound files used in the present study were not optimised to equalise their intelligibility. Since threshold SNRs tend to vary from digit to digit, this lack of optimisation likely flattened participants’ psychometric functions and added noise to the DIN threshold data. Optimisation following the methods of Akeroyd et al. (2015) or Smits et al. (2013) should enhance the precision of DIN threshold measurement and represents an important foundation for the use of the Arabic DIN test in hearing screening. The capacity of Arabic DIN data to predict hearing loss may ultimately be higher than suggested by the presently reported data, once digit intelligibility is equalised across the test material.

As discussed above, the study's sample size and characteristics did not permit adequate characterisation of the complex relations between age and Arabic antiphasic DIN data. Since age is associated not only with increasing PTA_4FA_ but also with different patterns, symmetries, and aetiologies of hearing loss and with independent effects on DIN performance, its influence on DIN data is likely multifarious. If age and DIN data are to be combined to provide effective remote hearing screening, more extensive supporting data may be required, to disentangle the complex relations between age, audiometric, and DIN data.

## Conclusion

Arabic antiphasic DIN thresholds are closely related to poorer-ear audiometric thresholds, after controlling for age. Crucially, they allow sensitive and specific identification of listeners with mild-or-greater hearing impairment. In all respects, Arabic diotic DIN thresholds perform less well. DIN data were obtained from a highly diverse sample of listeners, using browser-based DIN software designed for self-testing at home. Findings suggest that the browser-based Arabic antiphasic DIN may be of value in remote hearing screening. An additional step towards this goal may be the recruitment of a larger sample of listeners, to determine whether correction of DIN threshold cutoffs for age improves their sensitivity and specificity.

## Supplemental Material

sj-docx-1-tia-10.1177_23312165251320439 - Supplemental material for Arabic Digits-in-Noise Tests: Relations to Hearing Loss and Comparison of Diotic and Antiphasic Versions Supplemental material, sj-docx-1-tia-10.1177_23312165251320439 for Arabic Digits-in-Noise Tests: Relations to Hearing Loss and Comparison of Diotic and Antiphasic Versions by Adnan M. Shehabi, Christopher J. Plack, Margaret Zuriekat, Ola Aboudi, Stephen A. Roberts, Joseph Laycock and Hannah Guest in Trends in Hearing
