# Supplementary material for: Arabic Digits-in-Noise Tests: Relations to Hearing Loss and Comparison of Diotic and Antiphasic Versions
Source: Trends Hear. 2025 Mar 21;29:23312165251320439. doi: 10.1177/23312165251320439 (PMC11930467; doi:10.1177/23312165251320439)
Supplement: sj-docx-1-tia-10.1177_23312165251320439 - Supplemental material for Arabic Digits-in-Noise Tests: Relations to Hearing Loss and Comparison of Diotic and Antiphasic Versions [file sj-docx-1-tia-10.1177_23312165251320439.docx]

Supplementary material: Alternative analyses

# 1 Relations between DIN and better-ear PTA thresholds

The main paper is primarily concerned with *poorer-ear* PTA thresholds: how they relate to DIN thresholds and how well they are predicted by DIN thresholds. This focus is consistent with the pre-planned analyses laid out in the pre-registered protocol for the project.

However, some readers may be interested in relations with (and prediction of) better-ear PTA thresholds. Hence, we have repeated the main analyses, substituting better-ear PTA_4FA_ for poorer-ear PTA_4FA_:


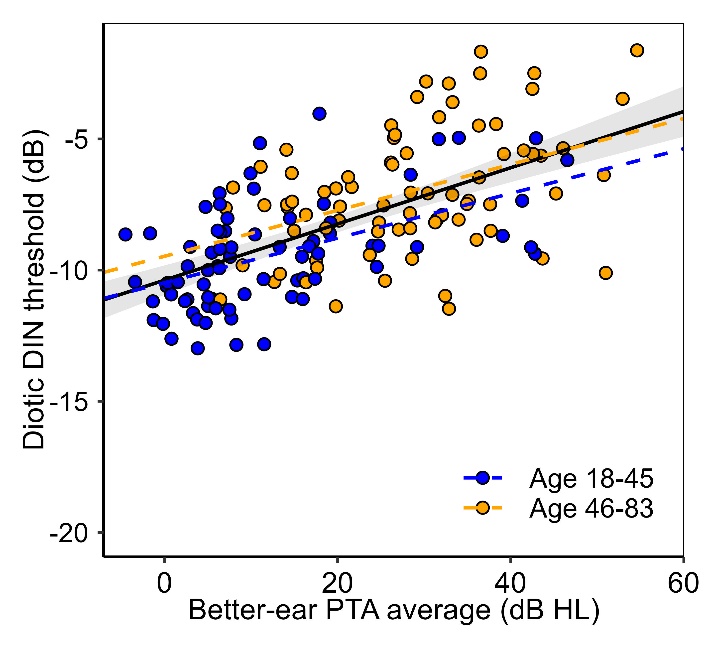

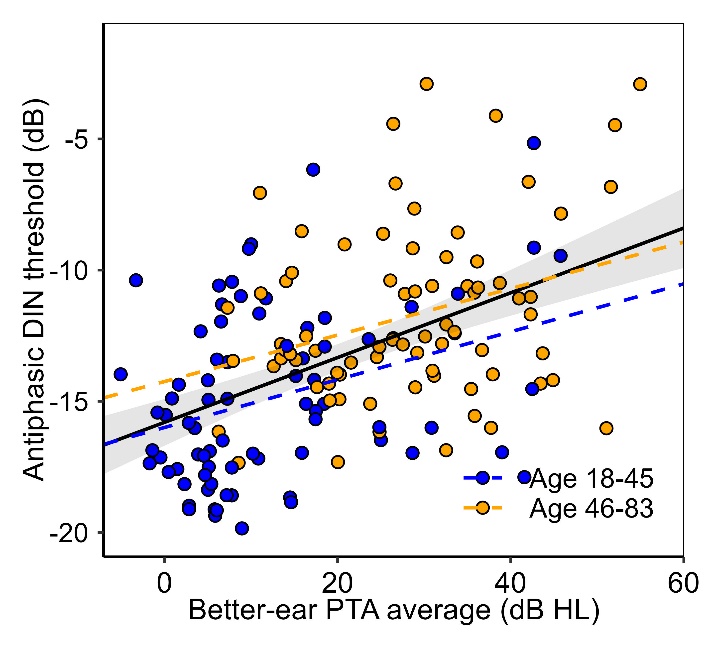


After adjusting for age, better-ear PTA_4FA_ is correlated with diotic DIN thresholds (*r* = 0.35, p < 0.0001) and antiphasic DIN thresholds (*r* = 0.22, *p* = 0.007). Formal comparison of the correlation coefficients via William’s t-test indicates a marginally stronger correlation for diotic than antiphasic stimuli (*p* = 0.04, two-tailed, without correction for multiple comparisons).

In a linear regression model (with DIN thresholds as the outcome variable and better-ear PTA_4FA_, test type, and the interaction between better-ear PTA_4FA_ and test type as predictors), the interaction term is non-significant (*p* = 0.45, two-tailed), indicating that test type (diotic versus antiphasic) has no significant effect on the steepness of the slope between better-ear PTA and DIN thresholds.

ROC curves were generated to quantify the performance of the diotic and antiphasic DIN in classifying various degrees of hearing loss. Estimates of AUC, sensitivity, and specificity are given in Table SM1. One-tailed DeLong’s tests were used to formally compare the AUCs (see Table SM2). Results suggest that the diotic DIN may be a better predictor of better-ear audiometric hearing loss than the antiphasic, though only at 20 and 25 dB HL is this difference associated with a p-value < 0.05.

| ***Table SM1*** | **Diotic DIN** | | | | | **Antiphasic DIN** | | | | |
| --- | --- | --- | --- | --- | --- | --- | --- | --- | --- | --- |
|  | ***AUC*** | ***SRT cutoff (dB)*** | ***Sensitivity*** | ***Specificity*** | ***Youden index*** | ***AUC*** | ***SRT cutoff (dB)*** | ***Sensitivity*** | ***Specificity*** | ***Youden index*** |
| **Detection of better-ear PTA_4FA_ >15 dB HL** | 0.78 | -8.0 | 0.64 | 0.74 | 0.38 | 0.72 | -13.0 | 0.59 | 0.70 | 0.29 |
|  |  | -8.5 | 0.72 | 0.64 | 0.35 |  | -13.5 | 0.63 | 0.61 | 0.23 |
|  |  | -9.0 | 0.80 | 0.58 | 0.37 |  | -14.0 | 0.71 | 0.56 | 0.27 |
| **Detection of better-ear PTA_4FA_ >20 dB HL** | 0.81 | -7.5 | 0.63 | 0.77 | 0.40 | 0.74 | -12.5 | 0.58 | 0.76 | 0.34 |
|  |  | -8.0 | 0.72 | 0.71 | 0.43 |  | -13.0 | 0.67 | 0.69 | 0.36 |
|  |  | -8.5 | 0.79 | 0.61 | 0.40 |  | -13.5 | 0.70 | 0.61 | 0.31 |
| **Detection of better-ear PTA_4FA_ >25 dB HL** | 0.83 | -7.0 | 0.61 | 0.87 | 0.48 | 0.75 | -12.0 | 0.54 | 0.78 | 0.33 |
|  |  | -7.5 | 0.68 | 0.76 | 0.45 |  | -12.5 | 0.63 | 0.75 | 0.39 |
|  |  | -8.0 | 0.77 | 0.70 | 0.47 |  | -13.0 | 0.72 | 0.68 | 0.40 |
| **Detection of better-ear PTA_4FA_ >40 dB HL** | 0.76 | -6.5 | 0.65 | 0.80 | 0.45 | 0.72 | -11.0 | 0.59 | 0.76 | 0.35 |
|  |  | -7.0 | 0.71 | 0.74 | 0.44 |  | -11.5 | 0.65 | 0.73 | 0.38 |
|  |  | -7.5 | 0.76 | 0.64 | 0.41 |  | -12.0 | 0.65 | 0.70 | 0.35 |

#

| ***Table SM2*** | **Diotic AUC** | **Antiphasic AUC** | **DeLong’s *Z*** | ***p*** |
| --- | --- | --- | --- | --- |
| **Detection of better-ear PTA_4FA_ >15 dB HL** | 0.78 | 0.72 | 1.36 | 0.09 |
| **Detection of better-ear PTA_4FA_ >20 dB HL** | 0.81 | 0.74 | 1.85 | 0.03 |
| **Detection of better-ear PTA_4FA_ >25 dB HL** | 0.83 | 0.75 | 2.06 | 0.02 |
| **Detection of better-ear PTA_4FA_ >40 dB HL** | 0.76 | 0.72 | 0.68 | 0.25 |

#

# 2 Relations to DIN BILD

The DIN binaural intelligibility level difference (BILD) is calculated as diotic DIN threshold minus antiphasic DIN threshold. After adjusting for age, DIN BILD is negatively correlated with poorer-ear PTA_4FA_ (*r* = -0.46, *p* < 0.0001) but not with better-ear PTA_4FA_ (*r* = 0.01, *p* = 0.92).


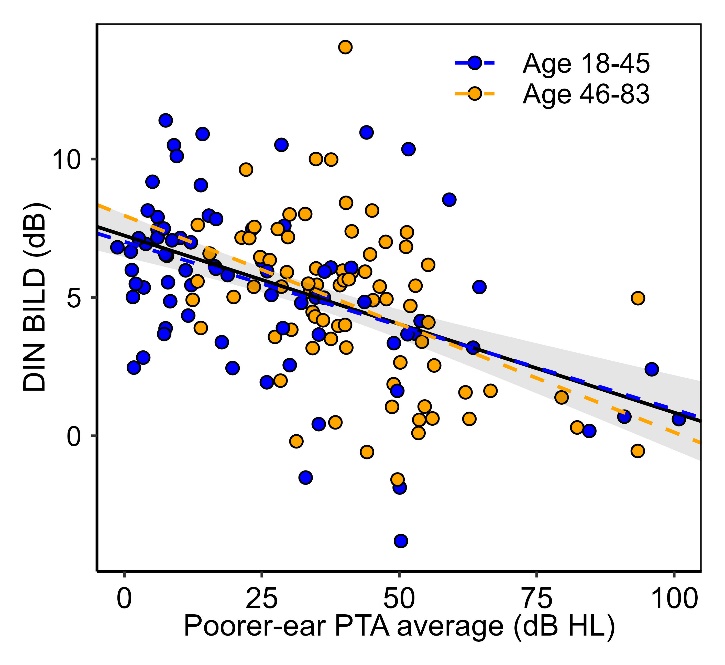

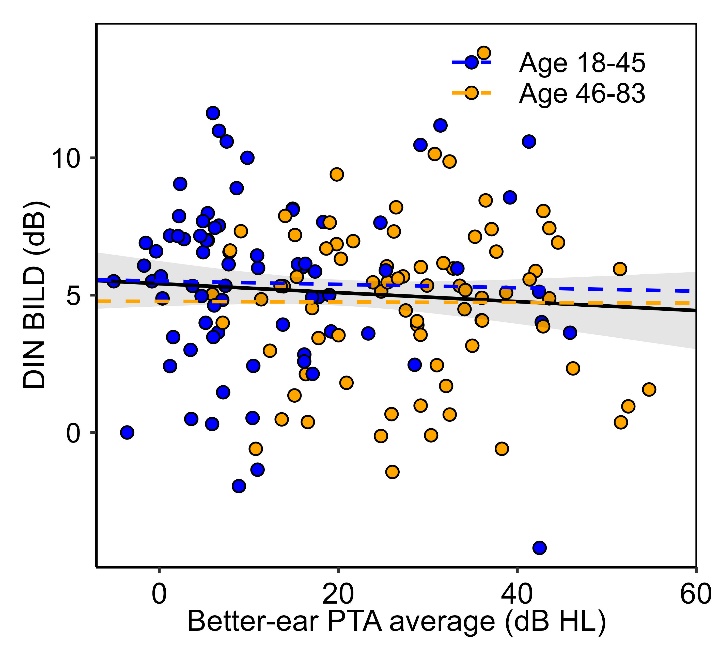


# 3 Effects of age, sex, and educational attainment

Effects of various demographic factors on diotic and antiphasic DIN thresholds were explored via the following multiple linear regression models:

Diotic DIN threshold ~ PTA_4FA_ + age + sex + educational attainment

Antiphasic DIN threshold ~ PTA_4FA_ + age + sex + educational attainment

*(Note that educational attainment is a numeric variable on a 1-5 scale, where 1 = completion of primary or middle school, 2 = completion of high school, 3 = post-school non-degree qualification, 4 = Bachelor’s degree, and 5 = postgraduate degree.)*

Diotic DIN thresholds were significantly related to age (*β* = 0.07 dB per year, *p* < 0.0001), marginally related to PTA_4FA_ (*β* = 0.02 dB per dB, *p* = 0.048), not significantly related to sex (*β* = 0.07 dB lower in women than in men, *p* = 0.83), and not significantly related to educational attainment (*β* = -0.17 dB per step up in educational level, *p* = 0.17).

Antiphasic DIN thresholds were significantly related to age (*β* = 0.05 dB per year, *p* < 0.0001), significantly related to PTA_4FA_ (*β* = 0.09 dB per dB, *p* < 0.0001), not significantly related to sex (*β* = 0.79 dB lower in women than in men, *p* = 0.07), and not significantly related to educational attainment (*β* = -0.27 dB per step up in educational level, *p* = 0.10), though note the non-significant trends for lower DIN thresholds in women and in the more highly educated.

An important caveat, as discussed in the main paper, is that type of hearing loss may mediate relations between demographic factors and DIN thresholds, complicating interpretation of the above results. For example, the proportion of hearing-loss cases that are bilaterally sensorineural is greater in older participants than in the young. The sample size of the present study is not sufficient to disentangle intrinsic effects of demographic factors from those that are mediated by pattern, symmetry, and/or aetiology of hearing loss.

# 4 Results with outlier included

An alternative analysis was conducted with all participants included, not excluding the participant with a BILD of -6.5 dB. Results are consistent with those reported in the main paper.

After adjusting for age, poorer-ear PTA_4FA_ is correlated with antiphasic DIN thresholds (*r* = 0.50, *p* < 0.0001) but the corresponding correlation with diotic DIN thresholds is marginal (*r* = 0.17, *p* = 0.04). Formal comparison of the correlation coefficients via William’s t-test indicates a significant difference between them (*p* = 0.0001, one-tailed).

In a linear regression model (with DIN thresholds as the outcome variable and poorer-ear PTA_4FA_, test type, and the interaction between poorer-ear PTA_4FA_ and test type as predictors), the interaction term is significant (*p* < 0.0001, one-tailed), indicating that increasing poorer-ear PTA_4FA_ leads to steeper increases in antiphasic than diotic DIN thresholds.

ROC curve analysis yields the following AUCs:

|  | Detection of poorer-ear PTA_4FA_ >15 dB HL | Detection of poorer-ear PTA_4FA_ >20 dB HL | Detection of poorer-ear PTA_4FA_ >25 dB HL | Detection of poorer-ear PTA_4FA_ >40 dB HL |
| --- | --- | --- | --- | --- |
| Diotic DIN | 0.79 | 0.78 | 0.74 | 0.67 |
| Antiphasic DIN | 0.89 | 0.86 | 0.85 | 0.79 |

# 5 Results with final 25 participants excluded

An alternative analysis was conducted with only the first 130 participants included, excluding the final 25 (recruited beyond our original recruitment target). Results are consistent with those reported in the main paper.

After adjusting for age, poorer-ear PTA_4FA_ is correlated with antiphasic DIN thresholds (*r* = 0.54, *p* < 0.0001) but the corresponding correlation with diotic DIN thresholds is marginal (*r* = 0.20, *p* = 0.03). Formal comparison of the correlation coefficients via William’s t-test indicates a significant difference between them (*p* < 0.0001, one-tailed).

In a linear regression model (with DIN thresholds as the outcome variable and poorer-ear PTA_4FA_, test type, and the interaction between poorer-ear PTA_4FA_ and test type as predictors), the interaction term is significant (*p* < 0.0001, one-tailed), indicating that increasing poorer-ear PTA_4FA_ leads to steeper increases in antiphasic than diotic DIN thresholds.

ROC curve analysis yields the following AUCs:

|  | Detection of poorer-ear PTA_4FA_ >15 dB HL | Detection of poorer-ear PTA_4FA_ >20 dB HL | Detection of poorer-ear PTA_4FA_ >25 dB HL | Detection of poorer-ear PTA_4FA_ >40 dB HL |
| --- | --- | --- | --- | --- |
| Diotic DIN | 0.80 | 0.78 | 0.73 | 0.65 |
| Antiphasic DIN | 0.85 | 0.82 | 0.84 | 0.78 |

# 
